# Role of bacteria in the production and degradation of *Microcystis* cyanopeptides

**DOI:** 10.1002/mbo3.343

**Published:** 2016-02-25

**Authors:** Enora Briand, Jean‐François Humbert, Kevin Tambosco, Myriam Bormans, William H. Gerwick

**Affiliations:** ^1^ UMR CNRS 6553 ECOBIOUniversity of Rennes 1Rennes Cedex35042France; ^2^ Center for Marine Biotechnology and BiomedicineScripps Institution of OceanographyUniversity of California San DiegoLa JollaCalifornia92093; ^3^ INRAiEES ParisUPMCParisFrance; ^4^ Skaggs School of Pharmacy and Pharmaceutical SciencesUniversity of California San DiegoLa JollaCalifornia92093

**Keywords:** Associated bacteria, biodegradation, cyanopeptides, *Microcystis*, phycosphere

## Abstract

The freshwater cyanobacteria, *Microcystis* sp.*,* commonly form large colonies with bacteria embedded in their mucilage. Positive and negative interactions between *Microcystis* species and their associated bacteria have been reported. However, the potential role of bacteria in the production and degradation of cyanobacterial secondary metabolites has not been investigated. In this study, a *Microcystis*‐associated bacterial community was isolated and added to the axenic *M. aeruginosa* PCC7806 liquid culture. After 3 years of cocultivation, we studied the bacterial genetic diversity adapted to the PCC7806 strain and compared the intra‐ and extracellular concentration of major cyanopeptides produced by the cyanobacterial strain under xenic and axenic conditions. Mass spectrometric analyses showed that the intracellular concentration of peptides was not affected by the presence of bacteria. Interestingly, the produced peptides were detected in the axenic media but could not be found in the xenic media. This investigation revealed that a natural bacterial community, dominated by Alpha‐proteobacteria, was able to degrade a wide panel of structurally varying cyclic cyanopeptides.

## Introduction

In the natural freshwater environment, cyanobacteria are always associated with heterotrophic bacteria that become embedded in their mucilage (Whitton 1973; Brunberg 1999; Berg et al. 2009). This habitat, in which intense interactions between cyanobacteria and bacteria occur, is referred to as the “phycosphere” by analogy of the rhizosphere of plants (Bell and Mitchell 1972). Several studies have focused on the interplay occurring between freshwater cyanobacteria and their associated bacteria, including competition or exchange of nutrients (Steppe et al. 1996; Fuks et al. 2005; Yuan et al. 2009), inhibition (Paerl 1996; Rashidan and Bird 2001; Ozaki et al. 2008) or enhancement of cyanobacterial growth (Paerl 1996; Casamatta and Wickstrom 2000; Eiler et al. 2006), degradation of cyanobacterial toxins (Bourne et al. 2001; Rapala et al. 2005; Ho et al. 2012), and finally formation of aggregates (Shen et al. 2011). Free‐living and attached bacterial communities have been shown to be distinct (Shi et al. 2012; Parveen et al. 2013) and marked changes occur in the structure and composition of attached bacterial communities during the course of a *Microcystis* bloom (Parveen et al. 2013), suggesting that the physiological status of cyanobacteria could have direct impacts on the associated bacterial community. Using a metatranscriptomic approach, Penn et al. (2014) recently highlighted the importance of functionally active co‐occurring bacteria in the metabolism of cyanobacterial exudates which can in turn help sustain cyanobacterial growth by nutrient and energy recycling.

It is well‐known that cyanobacteria release by exudation or cell lysis a variety of organic molecules such as organic acids, carbohydrates, proteins, and lipids (Amemiya et al. 1990; Tonietto et al. 2014; Kehr and Dittmann 2015), including bioactive compounds (Sivonen and Börner 2008). Indeed, freshwater cyanobacteria are able to produce a variety of secondary metabolites (aeruginosins: Ersmark et al. 2008; anabaenopeptins: Itou et al. 1999; cyanobactins: Sivonen et al. 2010; cyanopeptolins: Bister et al. 2004; microginins: Ishida et al. 2000; microviridins: Ishitsuka et al. 1990) displaying various bioactivities including cytotoxic, antiviral, antimalarial, or allelopathic through the inhibition of vital eukaryotic enzymes (mostly serine proteases; Welker and Von Döhren 2006; Niedermeyer 2015 and references herein). Among them, the microcystins (MC) are the most widely distributed and studied cyanotoxins due to their detrimental impact on a variety of organisms, including humans (Carmichael 1992). Although much is known about MC‐degrading bacteria (for reviews see Edwards and Lawton 2009; Ho et al. 2012; Dziga et al. 2013; Kormas and Lymperopoulou 2013), there have been very few studies on the potential role of bacteria in the degradation of the other cyanobacterial secondary metabolites (Kato et al. 2007).

In this study, we investigated the model organism *Microcystis aeruginosa* by high‐throughput sequencing, targeting a fragment of the 16S rRNA gene, combined with mass spectrometry techniques (LC–MS/MS), to examine the role of bacteria in the production and degradation of cyanopeptides. Our aims were (1) to compare the intra‐ and extracellular concentrations of major cyanopeptides produced by a *M. aeruginosa* strain in coculture with or without the natural bacterial community, (2) to characterize the diversity of a natural *Microcystis*‐associated heterotrophic bacterial community adapted to a *M. aeruginosa* strain culture, and (3) to explore specific interactions between cyanobacteria/bacteria within the phycosphere through the production of cyanopeptides and their degradation by the associated bacteria.

## Material and Methods

### Cyanobacterial and bacterial culture condition

The freshwater *M. aeruginosa* PCC 7806 cyanobacterial strain used was purchased from the Pasteur Culture collection of Cyanobacteria (http://cyanobacteria.web.pasteur.fr/). The interest to use this model was the possibility to work under axenic condition. PCC strains are the only axenic strains available in the world. The culture was cultivated in BG11_0_ medium supplemented with 1.8 mmol L^‐1^ of NaNO_3_ and 10 mmol L^‐1^ of NaHCO_3_ (hereafter modified BG11_0_ medium, Rippka and Herdman 1992). The culture grown under a 12:12 h light:dark regime using daylight white fluorescent tubes (Toshiba, 15 W, FL15D) with 10 *μ*mol m^−2^ sec^‐1^ illumination at a constant temperature of 25°C on an orbital shaker (90–100 rpm). The cultures were maintained in exponential growth phase by repeated dilution every 3 weeks in fresh culture medium, while the axenicity was regularly evaluated as described in Briand et al. (2012).

The natural bacterial community was isolated from the mucilage of *M. aeruginosa* colonies during a bloom in a French pond in October 2011 as described by Shen et al. (2011). Briefly, colonies were repeatedly washed in sterile Milli‐Q water and centrifuged (10 min, ×4000*g* at 4°C). The supernatant containing the natural bacterial community was filtered through sterile GF/C filter papers (Whatman, Buckinghamshire, UK) in order to avoid contamination by naturally occurring *M. aeruginosa* cells. Inoculation of 1 mL of the supernatant containing the natural bacterial community was immediately added to 40 mL of the axenic *M. aeruginosa* PCC 7806 culture. The xenic culture was scaled up with fresh modified BG11_0_ medium under the conditions described above to obtain a culture volume of 1 L. The xenic culture was maintained during 3 years by addition of fresh medium every 4–5 months to maintain a constant volume due to the loss by evaporation.

### Experiments setup

Four different treatments were established: an axenic *Microcystis* culture (*M. aeruginosa*_axenic_), a xenic *Microcystis* culture (*M. aeruginosa*_xenic_), a pure culture of heterotrophic bacteria (Bact), and an axenic *M. aeruginosa* cell‐free filtrate (*M. aeruginoa*_filtrate_). Each treatment was grown in 500‐mL Erlenmeyer flasks with a final volume of 300 mL. For the axenic and xenic treatments, 150 mL of exponentially growing axenic *M. aeruginosa* PCC 7806 strain were harvested by centrifugation (10 min, 4000*g* at 25°C) and transferred to 300 mL of modified BG11_0_ medium for the axenic treatment, or transferred to a volume of 300 mL consisting of equal volumes of the natural bacterial community and 2× concentrated culture modified BG11 medium for the xenic treatment. The volume of the natural bacterial community added to the axenic *M. aeruginosa* PCC 7806 culture was harvested from the xenic culture maintained during 3 years in exponential growth phase after centrifugation (10 min, 4000*g* at 25°C) and filtration through sterile GF/C paper filter (Whatmann, UK). “Bact treatment” consisted of 150 mL of bacterial culture (harvested as described above) and 150 mL of cell‐free filtrate of the axenic *M. aeruginosa* PCC 7806 culture. *M. aeruginosa* PCC 7806 cell‐free filtrate was obtained from an exponential growing axenic cyanobacterial culture after centrifugation (10 min, 4000*g* at 25°C) and filtration through sterile 0.2 *μ*m nitrocellulose filter (Millipore, Cork, Ireland). Treatment *M. aeruginosa*_filtrate_ corresponded to 300 mL of axenic cyanobacterial filtrate. Each treatment was prepared in triplicate and was incubated under the conditions described above. The experiment lasted 3 weeks at which time the cultures were harvested for extraction and High‐performance liquid chromatography mass spectrometry (HPLC–MS/MS) analysis.

### Sampling, extraction, and internal standard addition

After 3 weeks of experiment, volumes were harvested and centrifuged (10 min, 4000*g* at 25°C). Freeze‐dried biomass was extracted up to three times in 2:1 dichloromethane:methanol (DCM:MeOH, JT Baker, Center Valley, PA) and were evaporated to dryness. The supernatants were filtered through a sterile 0.2 *μ*m nitrocellulose filter (Millipore) and extracted on SPE‐C18 cartridges (GracePure^TM^; 5000 mg, Columbia, MD). Samples were evaporated to dryness under N_2_ and kept frozen until analysis.

Dry intra‐ and extracellular extracts were dissolved with acetonitrile (ACN, EMD Chemicals, Gibbstown, NJ) and internal standard (BOC‐L‐protected Ornithine, 0.25 mg mL^−1^ in ACN, Chem‐Impex International, Wood Dale, IL) was added before HPLC–MS/MS analysis.

### High‐performance liquid chromatography–electrospray ionization‐mass spectrometry (HPLC–ESI‐MS/MS) analysis

Each sample (20 *μ*L) was injected twice into a reverse‐phase HPLC system using a Phenomenex Kinetex C18 column (5*μ*, 100 mm × 4.60 mm) with a gradient of 5–99% ACN in water with 0.1% formic acid over 12 min, held at 99% for 5 min, and then returned to 5% at 22 min for another 3 min. The solvents were LC–MS grade (JT Baker, Center Valley, PA). The flow rate was 0.7 mL min^‐1^.

The HPLC eluate was electrospray ionized (capillary temperature at 325°C, source voltage at 5 kV, and a sheath gas flow rate of 69 L min^‐1^) and analyzed in the positive mode in the mass range of *m/z* from 300 to 2000 using a Thermo‐Finnigan LCQ Advantage ion trap mass spectrometer (Thermo‐Finnigan, San Jose, CA). MS/MS spectra were obtained in a data‐dependent manner using collision‐induced dissociation at 35 eV.

### Cyanopeptide identification and relative quantification

Secondary metabolites profiles of the *M. aeruginosa* PCC 7806 strain has been described in a previous experiment (Briand et al. 2015). Among those identified cyanopeptides, eight major compounds belonging to three known peptide classes were screened (Table 1): cyanobactins (Aer A, B, C, and D), cyanopeptolins (Cya A and Cya 963A), and microcystins (MC‐LR and Des‐MCLR).

**Table 1:** Peptides produced by the studied strains

Peptide class	*m/z* [M+H]^+^	Peak number (retention time in min)	Assignment (Reference)
Microcystin (MC)	981	2 (11.1)	Des‐MCLR (Mayumi et al. 2006)
	995	3 (11.2)	MC‐LR (Mayumi et al. 2006)
Cyanopeptolin (Cya)	946 [M‐H_2_O]^+^	5 (13.0)	Cyanopeptolin 963A (Bister et al. 2004)
	957	4 (11.6)	Cyanopeptolin A (Martin et al. 1993)
Cyanobactin (Aer)	517	7 (14.3)	Aerucyclamide C (Portmann et al. 2008b)
	533	8 (14.6)	Aerucyclamide B (Portmann et al. 2008a)
	535	6 (14.0)	Aerucyclamide A (Portmann et al. 2008a)
	603 [M+OH]^+^	1 (10.7)	Aerucyclamide D (Portmann et al. 2008b)

In order to compare relative concentrations of specific metabolites between different treatments, the peak area was determined with the software Xcalibur (Thermo) using the Peak Detection Genesis Algorithm. The quantification was based on the ratio of the peak area of metabolites and the added internal standard, BOC‐L‐protected ornithine. Data were then normalized to the dry weight for intracellular metabolites and to the volume for extracellular metabolites as described in Winnikoff et al. (2014).

### DNA extraction and high‐throughput sequencing

The natural bacterial community was sequenced after 3 years of coculture with the *M. aeruginosa* strain. The cell pellet of the xenic culture was centrifuged (10 min, 4000 *g*, 25°C), freeze‐dried overnight, and kept frozen until DNA extraction. The DNA extraction procedure was based on mechanical and chemical extraction and was adapted from the procedure described in Massana et al. (1997). A fragment of the 16S rRNA gene, including the variable V4–V5 region, was amplified by PCR from the DNA using primers 515F (5′‐GTGYCAGCMGCCGCGGTA‐3′) and 909R (5′‐CCCCGYCAATTCMTTTRAGT‐3′) (Tamaki et al. 2011). The sequencing was performed by Research and Testing Laboratory (Lubbock, Texas) on IlluminaMiSeq. The forward and reverse reads were merged together using the PEAR Illumina paired‐end read merger (Zhang et al. 2014). Chimera detection and removal by executing UCHIME (Edgar et al. 2011) and sequences were clustered at a 4% divergence into OTUs using the UPARSE algorithm (Edgar 2013). The centroid sequence from each cluster was then run against either the USEARCH global alignment algorithm (Edgar 2010) or the RDP Classifier against a database of high‐quality sequences derived from the NCBI database.

### Statistical analysis

Statistical differences in normalized relative concentrations were evaluated using two‐tailed *t*‐test. The analyses were conducted with the software Graphpad Prism 4.00 (San Diego, CA). Differences were accepted as statistically significant when *P *<* *0.05. Values are given as mean ± standard deviation (SD).

## Results and Discussion

### Comparison of the intra‐ and extracellular concentrations of major cyanopeptides produced by *M. aeruginosa* under xenic and axenic conditions

In a first experiment, we compared the secondary metabolic profile of the axenic *M. aeruginosa* strain PCC 7806 with the profile of the same strain after the addition of a natural bacterial community. As described in a previous experiment (Briand et al. 2015), the axenic strain produced eight major compounds (Table 1) belonging to three known peptide classes: cyanobactins (Aer A, B, C, and D), cyanopeptolins (Cya A and 963A), and microcystins (MC‐LR and Des‐MCLR).

**Figure 1:**
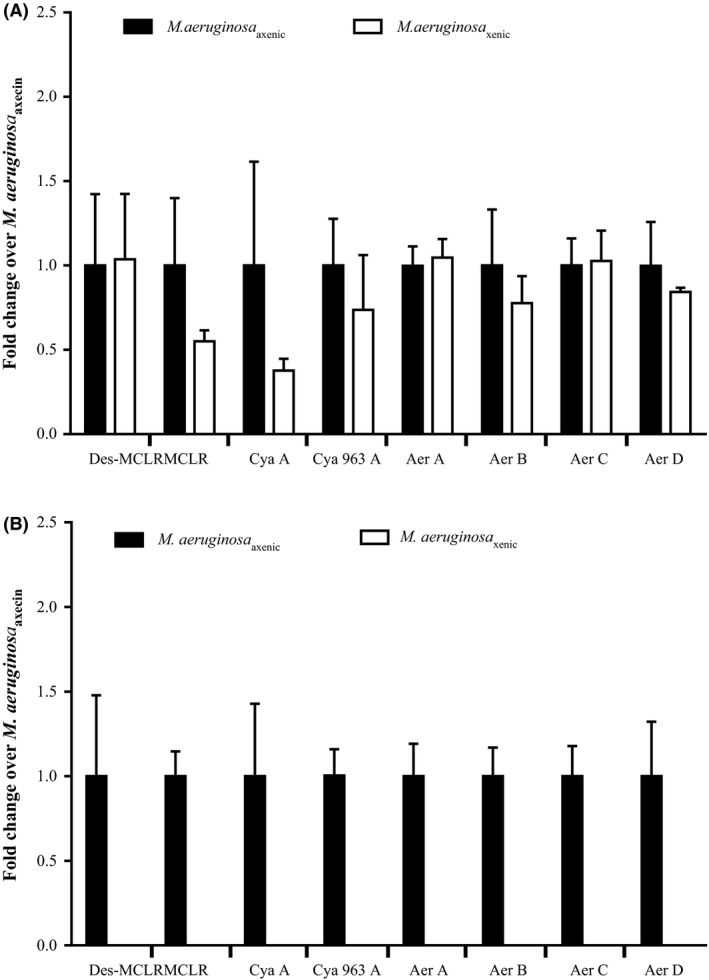
Relative concentrations of the main cyanopeptides produced by *M. aeruginosa* PCC 7806 strain (A: intracellular and B: extracellular) under axenic (black histograms) or xenic conditions (white histograms). Aer: Aerucyclamide, Cya: Cyanopeptolin, Des‐MCLR: Desmethyl Microcystin LR, MC‐LR: Microcystin LR.

No significant difference was found in the intracellular concentration of the eight cyanopeptides of the axenic and xenic PCC 7806 strain (Fig. 1A, P values >0.05), suggesting that the associated bacteria do not directly influence the production of secondary metabolites by *M. aeruginosa* PCC 7806. Conflicting findings have been reported concerning this bacterial–cyanobacterial interaction in previous studies. Sivonen (1990) has shown that MC production by *Planktothrix agardhii* strains was not influenced by the presence of bacteria whereas Dziallas and Grossart (2011) reported a strong impact of heterotrophic bacteria on the quantity and nature of MC variants produced by a *M. aeruginosa* strain. It has also been widely reported that bacteria influence the metabolic profile of marine diatoms; for example, the rate of biosynthesis of the neurotoxin domoic acid increases in xenic cultures of *Pseudo‐nitzschia multiseries* (Bates et al. 1995, 2004; Kobayashi et al. 2009; Stewart 2008). The regulation of production of such secondary metabolites, even for the most widespread and studied MC, is still unclear and requires further investigation concerning their regulation in response to biotic and abiotic conditions.

When comparing the extracellular fractions between the axenic and xenic strains, all the cyanopeptides present in the intracellular fraction were detected in the axenic media but they were completely absent in the xenic media (Fig. 1B). The first result provides evidence that the major cyanopeptides detected in the intracellular fraction are released out of the cells due to lysis and/or active transport. The second finding showed that degradation and/or transformation of these natural products by bacteria might cause the observed result, although we cannot rule out the possibility of inhibition of excretion by the bacterial community.

### Characterization of the natural bacterial community associated to *M. aeruginosa*

**Figure 2:**
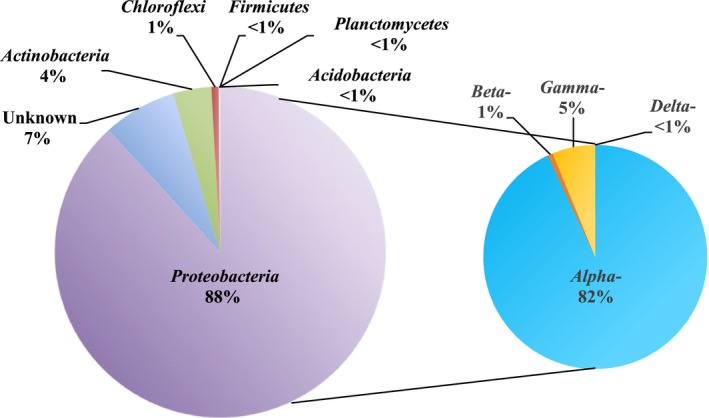
Relative abundance of major bacterial phyla.

In order to characterize the bacteria potentially involved in degrading these metabolites, we sequenced the bacterial community from the xenic PCC 7806 strain. This community was dominated by Proteobacteria (88%), in particular by Alpha‐proteobacteria (82%), and all other phyla were present at very low abundance (Fig. 2). The global structure of this community differs significantly from bacterial communities occupying freshwater lakes that are characterized by a codominance of Actinobacteria, and Alpha‐ and Betaproteobacteria (Humbert et al. 2009; Newton et al. 2011). However, Proteobacteria have previously been found to dominate *Microcystis*‐associated bacterial communities (Li et al. 2011; Shi et al. 2012; Parveen et al. 2013). Moreover, a recent metatranscriptomic study of *Microcystis*‐associated bacterial communities revealed the prevalence of Proteobacteria interacting closely with cyanobacteria as indicated by the highest transcription levels being observed for uptake of branched‐chain amino acids and organic phosphorus compounds (Penn et al. 2014). Interestingly, when looking at the OTU level, it appears that the dominant OTUs belong to the order of Rhizobiales (Table S1) display a high sequence similarity with OTUs found in the rhizosphere of plants and/or in environments displaying high quantities of organic matter (OM) such as sludge (*Agrobacterium tumefaciens*,* Rhizobium* sp., *Hyphomicrobium* sp., and *Mezorhizobium* sp.). Similarly, the only Actinobacteria OTU found displays a high sequence similarity with *Rhodococcus erythropolis*, which is known for its ability to degrade organic pollutants (De Carvalho et al. 2014). The genus *Sphingomonas,* accounting for 2% of the total bacterial diversity in this study, has been pointed by most studies to be closely associated with *Microcystis* (Shi et al. 2009, 2012; Dziallas and Grossart 2011; Shen et al. 2011; Zhu et al. 2014). Sphingomonadales species are well adapted to the cyanobacterial phycosphere, especially that of *Microcystis* with regard to their capacity to degrade cyanobacterial toxins and other problematic organic compounds (Wilkes et al. 1996; Zylstra and Kim 1997; Berg et al. 2009). With this regard, the bacterial community added to the xenic *Microcystis* strain is most likely adapted to degrade cyanobacterial‐derived OM exudates including cyanopeptides.

### Cyanopeptides‐degradation capability of the natural bacterial community associated to *M. aeruginosa*

**Figure 3:**
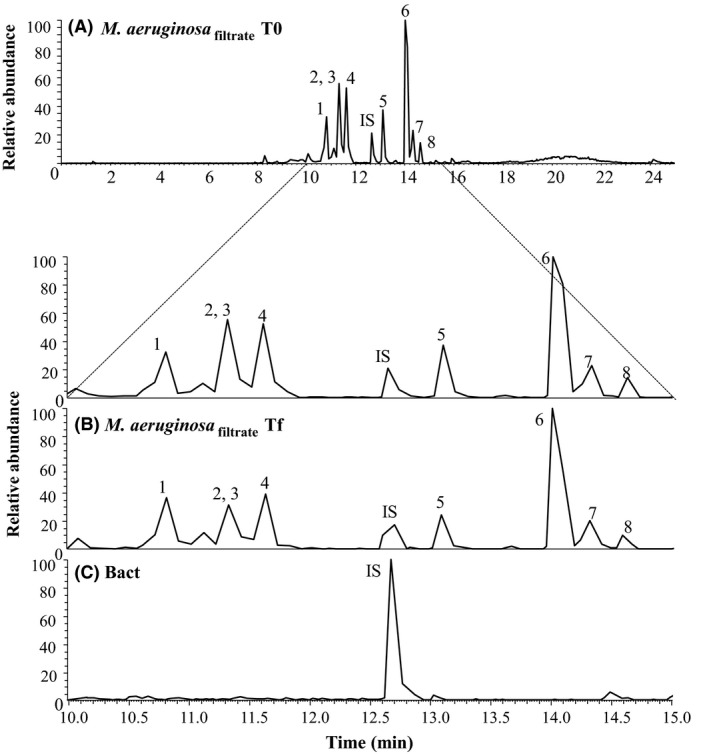
Chromatograms obtained for the extracellular cell‐free filtrate of the axenic *M. aeruginosa* PCC 7806 strain (*M. aeruginosa*_filtrate_) at the beginning (A) and at the end of the experiment (B), and that obtained for a pure culture of heterotrophic bacteria (Bact) treatment (C). Peaks legend: 1‐Aer D, Aerucyclamide D; 2‐Des‐MCLR, Desmethyl Microcystin LR; 3‐MC‐LR, Microcystin LR; 4‐Cya A, Cyanopeptolin A; 5‐Cya 963A, Cyanopeptolin 963A; 6‐Aer A, Aerucyclamide A; 7‐Aer C, Aerucyclamide C; 8‐Aer B, Aerucyclamide B; IS, Internal Standard.

With the goal to confirm the degradation of cyanopeptides by bacteria, we added the filtrate of the axenic PCC 7806 strain to two flasks either containing or not the natural bacterial community identified above (Fig. 3A).

Cyanopeptides were detected at the beginning of the experiment in the cell‐free filtrate of the axenic strain (Fig. 3A) and also after 3 weeks in the flasks without a bacterial community (Fig. 3B). On the other hand, chromatograms obtained for the extracellular cell‐free filtrate of the bacterial pure culture (Fig. 3C) were completely flat except for the peak corresponding to the internal standard. None of the eight compounds were detected by scanning chromatograms for their molecular ion masses at their respective retention time, suggesting that the natural bacterial community was able to completely remove the cyanopeptides to concentrations below that required for analytical detection. To our knowledge, this is the first study showing that a natural bacterial community is able to degrade not only MC but also other cyanobacterial secondary metabolites (cyanopeptolins and aerucyclamides in this study). Of all the cyanotoxins biodegradation studies, most have focused on the MC, as from a practical perspective, such as cyanotoxin‐degrading bacteria could be implemented as a low‐cost biological treatment option in water or sewage treatment plants. So far, the majority of isolated organisms reported as having the ability to degrade MC or other cyanotoxins appears to belong to the class Alpha‐proteobacteria and members of the Sphingomonadaceae are the most prevalent family among this class (for reviews see Edwards and Lawton 2009; Ho et al. 2012; Dziga et al. 2013; Kormas and Lymperopoulou 2013). Kato et al. (2007) showed the more or less effective degradation of different cyclic cyanopeptides (anabaenopeptin A, aeruginopeptin 95A, microcyclamide, MC‐LR, microviridin I, nodularin, and nostophycin) by cell extracts of the *Sphingomonas* sp. B‐9, first isolated for its MC‐degradation ability (Imanishi et al. 2005). In this study, *Shingomonas* sp. accounted for only 2% of the total bacterial diversity suggesting that several species of the natural bacterial community were probably also involved in the degradation of the full spectrum of naturally occurring peptides. Further studies using the RNA stable isotope probing (RNA‐SIP, Manefield et al. 2002) technique would be an effective approach for specifically identifying active microorganisms that assimilate particular ^13^C‐labeled substrates (*e.g*., cyanobacterial cell extracts or specific cyanopeptides) into their cellular biomass. Such knowledge would be instrumental in employing their cyanopeptide‐degrading capability for bioremediation, reducing the costs associated with water or environmental treatments.

Therefore, our results demonstrate that the bacterial community associated with a *Microcystis* bloom and exposed to *Microcystis* exudates was able to degrade effectively dissolved cyanopeptides as one of its DOC and nitrogen sources required for growth. Interestingly, the growth of xenic *Microcystis* cultures was self‐sufficient over 3 years (fresh medium was added every 4–5 months to maintain a constant volume due to the loss by evaporation), whereas the axenic culture needed repeated dilution every 3 weeks in fresh culture medium in order to be maintained in the exponential growth phase. Although we did not measure the bacterial growth, our observations suggest that *Microcystis* exudates containing dissolved cyanopeptides were able to provide the bulk of dissolved organic carbon (DOC) and nutrients needed for bacterial growth. The impact of DOC released by cyanobacteria on growth and activity of associated bacteria has been shown in previous studies (Robarts and Zohary 1986; Christoffersen et al. 2002; Kirkwood et al. 2006). In turn, the bacterial community may contribute to support cyanobacterial growth by remineralizing this portion of the dissolved organic matter (DOM) back to CO_2_ (Cho and Azam 1988). Coupling isotopic tracers with imaging mass spectrometry analysis (NanoSIMS) would be a powerful approach to highlight and quantify the relative role of cyanopeptides among the pool of DOM in providing carbon and nutrients from cyanobacteria to heterotrophic bacteria and vice versa. Previous studies have been performed to visualize and quantify the nitrogen transfer from N_2_‐fixing symbionts to their hosts [*e.g.,* bacteria/shipworm symbiosis (Lechene et al. 2007) and cyanobacteria/diatom symbiosis (Foster et al. 2011)], or more recently, sulfur cycling within a bacterial consortium (Wilbanks et al. 2014). The isotope tracer/NanoSIMS approach would also provide valuable information on the degradation rate of cyanopeptides by associated bacteria and on the flux of cyanopeptide‐derived carbon and nutrients within the cyanobacterial phycosphere. Hence, mutualistic interactions through the production, degradation, and remineralization of cyanopeptides within the tightly coupled microbial consortia may contribute to the ecological success of *Microcystis* in freshwater ecosystems.

## Conflict of Interest

The authors declare no conflict of interest.

## Supporting information


**Table S1.** Relative abundance at the OTU level.
