# Supplementary material for: Role of bacteria in the production and degradation of Microcystis cyanopeptides
Source: Microbiologyopen. 2016 Feb 25;5(3):469–78. doi: 10.1002/mbo3.343 (PMC4905998; doi:10.1002/mbo3.343)
Supplement: Supplementary file 1 — Table S1. Relative abundance at the OTU level. [file MBO3-5-469-s001.docx]

**Supplementary information**

**Table S1:** Relative abundance at the OTU level.

| **Phylum** | **Class** | **Group/Order** | **Family** | **Genus** | **OTU** | **%** |
| --- | --- | --- | --- | --- | --- | --- |
| *Acidobacteria* | *Acidobacteriia* | *Acidobacteriales* | *Acidobacteriaceae* | *Acidobacterium* | *Acidobacterium sp.* | 0.03 |
|  | *Holophagae* | *Holophagales* | *Holophagaceae* | *Holophaga* | *Holophaga sp.* | 0.03 |
| *Actinobacteria* | *Actinobacteria* | *Actinomycetales* | *Nocardiaceae* | *Rhodococcus* | *Rhodococcus erythropolis* | 3.60 |
|  |  |  | *Tsukamurellaceae* | *Tsukamurella* | *Tsukamurella sp.* | 0.14 |
| *Chloroflexi* | *Thermomicrobia* | *Sphaerobacterales* | *Sphaerobacteraceae* | *Sphaerobacter* | *Sphaerobacter sp.* | 0.72 |
| *Firmicutes* | *Bacilli* | *Bacillales* | *Staphylococcaceae* | *Staphylococcus* | *Staphylococcus aureus* | 0.03 |
| *Planctomycetes* | *Planctomycetia* | *Planctomycetales* | *Planctomycetaceae* | *Rhodopirellula* | *Rhodopirellula sp.* | 0.06 |
| *Proteobacteria* | *Alphaproteobacteria* | *Rhizobiales* | *Rhizobiaceae* | *Agrobacterium* | *Agrobacterium tumefaciens* | 20.34 |
|  |  |  |  | *Rhizobium* | *Rhizobium sp.* | 7.46 |
|  |  |  | *Hyphomicrobiaceae* | *Hyphomicrobium* | *Hyphomicrobium sp.* | 16.95 |
|  |  |  | *Phyllobacteriaceae* | *Mesorhizobium* | *Mesorhizobium sp.* | 12.33 |
|  |  |  | *Bradyrhizobiaceae* | *Bosea* | *Bosea sp.* | 5.92 |
|  |  |  |  | *Bradyrhizobium* | *Bradyrhizobium sp.* | 0.03 |
|  |  |  | *Hyphomicrobiaceae* | *Rhodomicrobium* | *Rhodomicrobium sp.* | 0.03 |
|  |  | *Caulobacterales* | *Caulobacteraceae* | *Brevundimonas* | *Brevundimonas sp.* | 10.48 |
|  |  | *Sphingomonadales* | *Sphingomonadaceae* | *Sphingomonas* | *Sphingomonas sp.* | 2.20 |
|  |  |  |  | *Sandaracinobacter* | *Sandaracinobacter sibiricus* | 1.73 |
|  |  | *Alphaproteobacteria* | *Alphaproteobacteria* | *Rasbo* | *Rasbo sp.* | 2.04 |
|  |  |  |  | *Ronia* | *Ronia tepidophila* | 0.55 |
|  |  | *Rhodospirillales* | *Acetobacteraceae* | *Roseomonas* | *Roseomonas stagni* | 1.51 |
|  |  |  |  |  | *Roseomonas lacus* | 0.44 |
|  |  |  |  | *Acidisphaera* | *Acidisphaera sp.* | 0.03 |
|  |  |  |  | *Gluconacetobacter* | *Gluconacetobacter sp.* | 0.03 |
|  | *Betaproteobacteria* | *Burkholderiales* | *Burkholderiaceae* | *Limnobacter* | *Limnobacter thiooxidans* | 0.69 |
|  | *Deltaproteobacteria* | *Myxococcales* | *Polyangiaceae* | *Byssovorax* | *Byssovorax sp.* | 0.03 |
|  | *Gammaproteobacteria* | *Chromatiales* | *Chromatiaceae* | *Nitrosococcus* | *Nitrosococcus sp.* | 5.45 |
| No hit |  |  |  |  |  | 7.18 |
